# Improving Adherence to National Laparoscopic Appendicectomy Documentation Guidelines: A Quality Improvement Project

**DOI:** 10.7759/cureus.94923

**Published:** 2025-10-19

**Authors:** Kayden Chahal, Valentina James, Charlotte Rowe, Isobel Thomas, Maro Paroikaki, Ahmed Elnewishy, Ahmed Moustafa, Charlotte Florance, Liam Poynter

**Affiliations:** 1 General Surgery, Maidstone and Tunbridge Wells, Tunbridge Wells, GBR; 2 Surgery, Maidstone and Tunbridge Wells, Tunbridge Wells, GBR; 3 General Surgery, East Sussex Hospital, East Sussex, GBR

**Keywords:** girft (getting it right first time), laparoscopic appendectomy, medical documentation, national guideline, quality improvement (qi)

## Abstract

Background

High-quality operative documentation is critical for clinical communication, patient safety, audit, education, and medico-legal protection. In 2022, the Get It Right First Time (GIRFT) initiative published best practice guidelines for laparoscopic appendicectomy documentation, detailing 44 recommended data points. This study aimed to improve adherence to these standards within a district general hospital through a three-cycle quality improvement project.

Materials and methods

A Plan-Do-Study-Act methodology was employed across three audit cycles (April 2024, September 2024, and January 2025). All laparoscopic appendicectomy cases meeting the inclusion criteria were assessed using a GIRFT-based audit tool. Interventions included a governance meeting, an in-theatre checklist (post-cycle one), and the introduction of an electronic operative note template aligned with GIRFT guidance (post-cycle two). Data were analyzed descriptively, and percentage improvements between cycles were calculated.

Results

Fifty-six operation records were analyzed (20 in cycle one, 18 in cycles two and three). Progressive improvements in documentation completeness were observed across most categories, with the most significant gains following electronic template implementation. By cycle three, multiple sub-categories, including appendix details, entry to abdomen, and operative prep, achieved 100% documentation rates (18/18). The only significant decline was in skin preparation documentation (-100%), due to a template omission.

Conclusions

Structured interventions, particularly an electronic operative note template aligned with GIRFT standards, substantially improved operative documentation quality. These low-cost, scalable changes could be replicated in other centers. Future work should assess long-term sustainability and explore parallel implementation of electronic consent templates.

## Introduction

Writing a high-quality operation note is the only true opportunity to provide a comprehensive account of the intraoperative course. Indeed, in the United Kingdom, clear and concise documentation of your work is a statutory duty as set out in "Good Medical Practice (2024)" [1]. Importantly, this guidance mentions legibility; the transition to digitalized operative notes is guaranteed to improve legibility rates to 100% [2]. In a surgical context, these records serve as a pivotal tool driving potentially life-changing clinical decision-making specific to each case [3]. Moreover, from a medicolegal perspective, meticulous documentation is one of the critical factors that aid defendants [4].

Another key document, particularly in the medicolegal context, is the surgical consent form. Proper documentation is an essential element of the informed consent process [5].

National-level analysis of national guidance, medical negligence claims, and statements from expert medical witnesses led to "Get it Right First Time" (GIRFT), the Association of Surgeons - Great Britain and Ireland (ASGBI), and the Royal College of Surgeons (RCS) England producing joint guidance in "Best Practice for Laparoscopic Appendicectomy Documentation" (September 2022) [6]. This guideline details 44 data points recommended as “gold standard” documentation for every laparoscopic appendicectomy operation note. The guideline recommends what would be “reasonably expected” to be documented to support good clinical communication with colleagues and potential review of operations. Of the 44, 12 points can be considered context dependent: these include catheter insertion and all points pertaining to the postoperative plan, apart from the signature.

Within our institution, we noted the incongruity of written documentation when reviewing postoperative patients. Concerning operative notes and consent forms, we initiated a quality improvement project with the following aims.

Our primary objective was to increase the proportion of operation notes meeting all mandatory elements of the "Best Practice for Laparoscopic Appendicectomy Documentation" guidelines to at least 90% by the third cycle. Secondary objectives were to improve awareness of context-specific subdomains and support good clinical communication with patients and colleagues.

## Materials and methods

This was a single-center quality improvement project carried out in a busy district general hospital surgical department (Maidstone and Tunbridge Wells NHS Trust). The hospital trust covers a population of approximately 760,000 and operates for individuals aged six and older. Inclusion criteria were simply all patients in the study period who had a laparoscopic appendectomy. Exclusion criteria were written notes deemed illegible by data collectors and procedures converted to open or incomplete data: missing operation note or consent form. The project was registered with the hospital governance team as per local guidelines. Formal ethical review was not sought for this service evaluation, as no patient-identifiable data was recorded.

Data collection consisted of three cycles, each consisting of three to four weeks of operations in April 2024, September 2024, and January 2025; cases were consecutive within the respective window. A data collection tool was built on the 44 data points identified in the GIRFT Best Practice statement [6] within an Excel spreadsheet (Microsoft Corp., Redmond, WA, USA) and utilized for all stages of data collection. An individual data collector was assigned to each cycle to eliminate inter-rater reliability. Data collectors were trained in information governance, all used the same tool, and were made fully aware of the GIRFT document. Data was collected retrospectively for the first loop and prospectively for the subsequent two. Sources included electronic patient records and written (paper-based) operative notes and consent forms for loops one and two, and typed (online) operative notes for loop three. In addition, note was made of the date of birth, date of admission, and date of operation; note was made of the cause for any significant delays to the procedure. It was pre-agreed that if any operation notes or consent forms were missing or illegible, that case would be excluded from analysis.

This project was conducted as a three-cycle audit using the Plan-Do-Study-Act (PDSA) methodology, following the GIRFT Best Practice guidance as the audit standard [6]. Interventions were implemented between each consecutive cycle. After loop one, a discussion was held to raise awareness in local clinical governance, prompting a push for the implementation of electronic operation notes. Additionally, a standardized “appendix documentation checklist” was displayed clearly in the confidential enquiry into perioperative death (CEPOD) theatre (Table 1). Between loops two and three, electronic operation notes were implemented, and a template (Table 2) was created incorporating the GIRFT guidance, which was strongly encouraged for use. Both the checklist and template were validated by two senior surgeons, experts in colorectal surgery. The data sets in each cycle were small and intended to guide local changes, so descriptive analysis was more appropriate than inferential statistics.

**Table 1:** Appendix documentation checklist displayed in the CEPOD theater above the stack of blank operation notes CEPOD: confidential enquiry into perioperative death, GIRFT: Get It Right First Time

Appendicectomy documentation
According to the GIRFT best practice document, the following details should be included:
In the consent form
Bowel resection
Conversion to open
(Specifically) laparotomy
Blood transfusion (tick box)
In the operation note
Name and grade of the surgical team and anaesthetic staff
Operation date and time
Peri-operative antibiotics
Patient positioning and skin preparation
Abdominal pressure
Findings, including the appendix position
Management of the appendix, including commenting on the base condition
Management of the appendicular artery and mesoappendix
Wash volume (if used)
Drain (if placed)
Appendicular extraction and whether sent for histology
Any hemostasis management and estimated blood loss
Closure technique
Postoperative instructions
Sign off with name, signature, and grade

**Table 2:** Elements of the operation note template implemented onto the the electronic notes system where surgeons would delete or input data as necessary pertaining to the operation AAST: American Association for the Surgery of Trauma

Laparoscopic appendicectomy operation note template	
Domain	Statement in template
Decision for surgery	Time/date was...
Urinary catheter	Yes OR no OR indwelling + size
Umbilical port	A 12 mm supra/infraumbilical was inserted under direct vision/cutdown OR Veress needle OR with optical port entry
Pneumoperitoneum	Established prior to insertion of the laparoscope
Ports	Working ports were placed under laparoscopic vision as follows: (infiltration of xx local anaesthesia to guide entry of) 5/10 mm (bladed/unbladed) port to LIF and 4/10 mm (bladed/unbladed) port to hypogastrium
Intra-abdominal pressure	(mmHg) was set to xx mmHg
Position	The patient was placed in the Trendelenburg position with the left side down
Laparoscopic findings	1. AAST grade I-V appendicitis - (qualify as) perforated/necrotic, pelvic/retro-caecal/retro-ileal/sub-phrenic/mid-abdomen, the presence of abscess, diffuse peritonitis, or macroscopically normal appendix
	2. Terminal ileum normal/abnormal
	3. Mesenteric adenopathy noted/not present
	4. Meckel’s yes/no
	5. Pelvic organs – right ovary/tube, left ovary/tube, pouch of Douglas, uterus mobile/not applicable
	6. Recto-sigmoid/sigmoid colon/presence of diverticular disease, etc.
	7. Gallbladder
Appendix location	The appendix was located in/at... and was easily/not easily identified
Appendiceal artery	Was skeletonised and divided by electrocautery/divided between (Ligaclips/Haem-Loks)
Mesoappendix management	The remainder of the mesoappendix was dissected, and the base of the caecum was (un)healthy and left intact - the junction of the appendix and caecum was clearly seen
Intraoperative complications	No
	Yes - including what action was taken to remedy them, including conversion from laparoscopic to open, any additional procedures performed, and the rationale for them
Appendix extraction	The (entire/piecemeal) appendix was placed in a sterile endoscopic bag for extraction via the umbilical port site and sent for histopathological analysis. The tissue sample was sent to pathology
Irrigation	Xx mL saline used for washout
Port removal	Haemostasis achieved. The ports were then removed under direct vision
Drain(s)	No
	Yes – size, type, via which port site, secure with silk suture
Closure	Fascia at 12 mm port site closed with 0 Vicryl. 3/0 subcuticular Monocryl to skin. Local anaesthetic used (type, %, volume)
Images	Taken during the procedure are attached to the operation record

## Results

A total of 56 records were included in the analysis: 20 in the initial cycle and 18 in the following two. The results of data collection for all cycles are displayed in Table 3. No records were excluded from analysis. There were three total delays of more than a day to the operation; the cause in all these cases was clinical priority due to a heavy emergency workload.

**Table 3:** Summary of the data collected across all three loops including percentage change in the intervals HDU: high dependency unit, ITU: intensive therapy unit, VTE: venous thromboembolism, N/A: not applicable

	Parameter assessed	Frequency of documentation in cycle 1 (N = 20)	% change after cycle 1	Frequency of documentation in cycle 2 (N = 18)	% change after cycle 2	Frequency of documentation in cycle 3 (N = 18)
Preoperative	Time of operative decision	20 (100%)	-11%	16 (89%)	13%	18 (100%)
	Clerking	19 (95%)	-18%	14 (78%)	21%	17 (94%)
	Result review	18 (90%)	-20%	13 (72%)	15%	15 (83%)
Consent	Conversion	19 (95%)	5%	18 (100%)	0%	18 (100%)
	Transfusion	12 (60%)	57%	17 (94%)	6%	18 (100%)
	Resection	12 (60%)	57%	17 (94%)	6%	18 (100%)
	Laparotomy	0 (0%)	0%	6 (33%)	100%	12 (67%)
Operating team	Surgeon name	18 (90%)	11%	18 (100%)	0%	18 (100%)
	Surgeon grade	1 (5%)	233%	3 (17%)	500%	18 (100%)
	Anaesthetist name	4 (20%)	67%	6 (33%)	167%	16 (89%)
	Anaesthetist grade	0 (0%)	0%	1 (6%)	1700%	18 (100%)
	Assistant name	18 (90%)	11%	18 (100%)	0%	18 (100%)
Operative prep	General anaesthetic	12 (60%)	39%	15 (83%)	20%	18 (100%)
	Operation date	17 (85%)	18%	18 (100%)	0%	18 (100%)
	Operation time	0 (0%)	0%	0 (0%)	N/A	18 (100%)
	Perioperative drugs	1 (5%)	233%	3 (17%)	267%	11 (61%)
	Catheter	4 (20%)	122%	8 (44%)	13%	9 (50%)
	Patient position	13 (65%)	37%	16 (89%)	6%	17 (94%)
	Skin preparation	6 (30%)	85%	10 (56%)	-100%	0 (0%)
Entry to the abdomen	Incisions	15 (75%)	19%	16 (89%)	13%	18 (100%)
	Open cut down (Hasson)	4 (20%)	94%	7 (39%)	157%	18 (100%)
	Pneumo established	8 (40%)	53%	11 (61%)	64%	18 (100%)
	Scope insertion under direct vision	5 (25%)	78%	8 (44%)	125%	18 (100%)
	Other port size	18 (90%)	11%	18 (100%)	0%	18 (100%)
	Other port position	18 (90%)	11%	18 (100%)	0%	18 (100%)
	Other ports under direct vision	11 (55%)	52%	15 (83%)	20%	18 (100%)
	Abdominal pressure	3 (15%)	85%	5 (28%)	260%	18 (100%)
Appendix details	Gross findings	17 (85%)	18%	18 (100%)	0%	18 (100%)
	Appendix location	6 (30%)	67%	9 (50%)	100%	18 (100%)
	Ligation technique	16 (80%)	25%	18 (100%)	0%	18 (100%)
	Appendix artery management	4 (20%)	67%	6 (33%)	200%	18 (100%)
	Mesoappendix management	15 (75%)	19%	16 (89%)	13%	18 (100%)
	Base condition	8 (40%)	53%	11 (61%)	64%	18 (100%)
End of operation	Wash volume	0 (0%)	0%	0 (0%)	N/A	18 (100%)
	Appendix extraction	14 (70%)	27%	16 (89%)	13%	18 (100%)
	Sent for histology	1 (5%)	233%	3 (17%)	467%	17 (94%)
	Haemostasis	6 (30%)	67%	9 (50%)	89%	17 (94%)
	Ports removed under direct vision	7 (35%)	59%	10 (56%)	70%	17 (94%)
	Intraoperative complications	6 (30%)	67%	9 (50%)	100%	18 (100%)
	Drain insertion	4 (20%)	67%	6 (33%)	-33%	4 (22%)
	Closure technique	16 (80%)	25%	18 (100%)	0%	18 (100%)
	Blood loss	3 (15%)	85%	5 (28%)	160%	13 (72%)
Postoperative plan	Antibiotics	13 (65%)	37%	16 (89%)	6%	17 (94%)
	Blood tests	1 (5%)	122%	2 (11%)	150%	5 (28%)
	HDU/ITU	0 (0%)	0%	0 (0%)	0%	0 (0%)
	Observations	1 (5%)	122%	2 (11%)	100%	4 (22%)
	Pathology	1 (5%)	122%	2 (11%)	100%	4 (22%)
	VTE prophylaxis	4 (20%)	67%	6 (33%)	67%	10 (56%)
	Eat and drink	16 (80%)	18%	17 (94%)	6%	18 (100%)
	Discharge	14 (70%)	27%	16 (89%)	0%	16 (89%)
	Removal of sutures	3 (15%)	85%	5 (28%)	260%	18 (100%)
	Follow-up	3 (15%)	85%	5 (28%)	0%	5 (28%)
	Photos	10 (50%)	56%	14 (78%)	-57%	6 (33%)
	Signature	18 (90%)	11%	18 (100%)	0%	18 (100%)

Preoperative documentation and consent

Both preoperative documentation and consent were unaffected by the introduction of the online pro forma. Nevertheless, the importance of accurate record-keeping was stressed at all stages. This is reflected in the equivocal trend of change in preoperative documentation with -11%, -18%, and -20%, which improved 13%, 21%, and 15%, respectively, to similar levels. There was an across-the-board uptrend in the quality of consent. By the third cycle, all consent forms included conversion to open, blood transfusion, and resection. Laparotomy did improve 100% from 6/18 (33%) to 12/18 (67%) from cycle two to three.

Operating team

Improvements were seen in the documentation of anesthetist names, with increases of 67% and 167%. Even larger improvements were made in anesthetist and surgeon grade; between cycle two and three, a 1700% and 500% increase was recorded. Surgeon and assistant grades were consistently well recorded, with either no difference or 11% at all stages.

Operative preparation

Documentation of the use of general anesthetic, operation date, and time all improved to 100% by loop three. In particular, the operative time went from not being recorded whatsoever to being documented in every case. Notably, the method of skin preparation was not documented in any of the three looped patients, representing a 100% decrease from the intermediate cycle.

Entry to the abdomen

All entries to the abdomen subcategories had perfect documentation, 18/18 (100%) by loop three. All subcategories improved in the two intervals. The most significant improvements were seen in the description of Hasson cut down, 94% and then 157%, and documentation of abdominal pneumoperitoneum pressure, 85% and then 260%.

Appendix details

All appendix details subcategories had perfect documentation 18/18 (100%) by loop three. Gross findings, ligation technique, and mesoappendix management were consistently well recorded with moderate improvements. Appendix location had a 67% and then a further 100% improvement; appendix artery management was 67% and then 200%, and base condition was 53% and then 64%.

End of operation

All end-of-operation documentation was either completely compliant, 18/18 (100%), or missing from one record, 17/18 (94%). Apart from drain insertion and blood loss, the latter still had an 85% and then a 160% improvement. If a drain was inserted, this was coded as recorded; however, this was due to intraoperative decision-making rather than quality of documentation. Every patient who had a drain had its insertion documented.

Postoperative plan

The postoperative plan documentation was the most variable of all the audited categories. This reflects the fact that patients receive tailored postoperative instructions based on their intraoperative findings, physiological status, specific factors, and surgeon preference. Of note, instructions to eat and drink (18% and 6%), removal of suture instructions (85% and 260%), and discharge timeline (27% and 0%) all demonstrated improvement; these were areas highlighted in clinical governance that all patients should have information recorded about.

## Discussion

High-quality operative documentation underpins effective clinical communication, continuity of care, audit, education, and medico-legal security. Our three-cycle quality improvement project effectively demonstrated that structured interventions can significantly enhance adherence to GIRFT best practice. Raising awareness, using visual prompts, and especially employing an electronic operative note template proved both practical and scalable. This is reflected in across-the-board improvements in all documentation categories by cycle three. All, apart from three, subcategories had either equal or improved documentation rates. The only relevant decrease in documentation was that of skin preparation (-100%). This highlighted an omission in the electronic template, which has since been patched.

During the initial cycle, handwritten notes and consent forms exhibited marked inconsistency across multiple data points. This finding aligns with earlier audits highlighting low completeness and variability in handwritten operative documentation [7]. For instance, in some centers, trainees’ handwritten notes recorded crucial elements such as surgery time, anesthetist name, and deep vein thrombosis prophylaxis in fewer than half of cases [8]. Similarly, broader audits have shown that few handwritten notes meet the full RCS documentation standards, with critical elements like estimated blood loss and complications frequently omitted [9].

The first intervention, a departmental governance discussion coupled with a checklist displayed in the theater, yielded modest improvement. The role of structured prompts and education in prompting behavior change is significant, though such passive interventions often fall short of achieving significant or sustained impact [7].

The most significant gains were seen with the second intervention: implementation of an electronic operative note template aligned with GIRFT guidance. Digitalization offers clear advantages: improved legibility, built-in prompts, and structured fields that reduce omissions. These findings mirror the Darent Valley Hospital study, a large UK audit of 405 operation notes, where electronic notes significantly outperformed handwritten ones in 17 of 18 RCS criteria and were 100% legible, compared to 91.7% of handwritten notes [10]. Other projects have demonstrated similar results; one surgical department saw operative notes 100% adherent to RCS criteria and eliminated delays following the introduction of an electronic template [11].

Beyond usability gains, complete and structured operative and consent documentation can potentially serve critical medico-legal functions. In litigation contexts, lapses in documentation can undermine legal defense and diminish trust, a risk mitigated when operations follow a standardized, comprehensive approach [12]. Thus, proper consent records are especially vital given evolving legal standards such as the Montgomery ruling, which demands that patients be informed of all material risks [13].

Despite these successes, our project has limitations. As a single-center study conducted within a district general hospital, with relatively small sample sizes, it may limit generalizability. Documentation improvements could have been influenced by observer bias or the Hawthorne effect, wherein clinicians’ awareness of ongoing evaluation alters their behavior. Finally, due to the time-limited nature of the study, there was little capacity to report downstream clinical outcomes or sustainability beyond the third cycle.

Nevertheless, our interventions were simple, low-cost, and replicable, and most of the limitations could be easily addressed in further re-audits. Generally, the success of the GIRFT-aligned electronic template suggests a scalable strategy that other trusts and surgical departments could adopt, contributing to national efforts to standardize documentation and enhance surgical outcomes. Although not directly investigated within the scope of this project, the potential outcomes resulting from standardized documentation, such as reduced medico-legal risk or improved information sharing, can be appreciated.

Future work should include evaluating the benefits of standardizing documentation and the long-term sustainability of documentation improvements, as well as exploring the use of electronic consent templates alongside operative notes. Moreover, evolving healthcare practices may include advanced technologies such as AI-assisted documentation, which has shown promise in improving accuracy and reducing clinician burden in other surgical contexts [14].

## Conclusions

This three-cycle quality improvement project demonstrated that introducing structured prompts and, most effectively, an electronic operative note template aligned with GIRFT guidance can markedly improve the quality and completeness of operative documentation. Such interventions are inexpensive, easily implemented, and scalable across surgical specialties. While some variability in certain categories reflects clinical tailoring, standardization of documentation enhanced clinical communication and medico-legal robustness. Broader adoption of GIRFT-aligned digital documentation, potentially integrated with electronic consent tools, may help achieve nationwide standardization and improved surgical outcomes.
